# Just Sentiments

**Published:** 1860-05

**Authors:** 


Just Sentiments.—The editor of the *American Medical Gazette*, in speaking of the "editorials" of a certain new journal, holds the following just sentiments, which cannot be too strongly recommended to the consideration of our confreres:—

"When he has been longer in the harness he will become more tolerant and forbearing, and will testify with Cotton Mather, that 'slander and detraction are sparks, which, if let alone, will go out of themselves;' and he will let his 'enemies' slide. If he expects to retain the position he holds without a conflict with unscrupulous rivals, he looks for better luck than his neighbors, for this is a tax which even laudable ambition must pay. In a few years more he will be *skinned* so often, that, like the eel, he will become *used to it*, and be less sensitive to assaults on himself when his editorial assaults on others will become moderated. 'Always use soft words, they cost nothing,' is the best motto for a journalist, *if* he can practice it."

